# Considerations for shared decision management in previously untreated patients with hemophilia A or B

**DOI:** 10.1177/20406207231165857

**Published:** 2023-04-17

**Authors:** Jan Astermark, Jan Blatný, Christoph Königs, Cédric Hermans, Victor Jiménez-Yuste, Daniel P. Hart

**Affiliations:** Department of Translational Medicine, Lund University, and Department of Hematology, Oncology and Radiation Physics, Skåne University Hospital, Jan Waldenströms gata 14, SE-205 02 Malmö, Sweden; Department of Pediatric Hematology, University Hospital Brno and Masaryk University, Brno, Czech Republic; Clinical and Molecular Hemostasis, Department of Pediatrics, University Hospital Frankfurt, Goethe University, Frankfurt, Germany; Hemostasis and Thrombosis Unit, Division of Hematology, Cliniques Universitaires Saint-Luc, Université catholique de Louvain (UCLouvain), Brussels, Belgium; Hematology Department, Hospital Universitario La Paz, Autónoma University, Madrid, Spain; The Royal London Hospital Haemophilia Centre, Barts and the London School of Medicine, QMUL, London, UK

**Keywords:** hemophilia, previously untreated patient, prophylaxis, therapy

## Abstract

Recent advances in therapeutics are now providing a wide range of options for adults and children living with hemophilia. Although therapeutic choices are also increasing for the youngest individuals with severe disease, challenges remain about early management decisions, as supporting data are currently limited. Parents and healthcare professionals are tasked with helping children achieve an inclusive quality of life and maintain good joint health into adulthood. Primary prophylaxis is the gold standard to optimize outcomes and is recommended to start before 2 years of age. A range of topics need to be discussed with parents to aid their understanding of the decisions they can make and how these will affect the management of their child/children. For those with a family history of hemophilia, prenatal considerations include the possibility of genetic counseling, prenatal investigations, and planning for delivery, together with monitoring of the mother and neonate, as well as diagnosis of the newborn and treatment of any birth-associated bleeding. Subsequent considerations, which are also applicable to families where infant bleeding has resulted in a new diagnosis of sporadic hemophilia, involve explaining bleed recognition and treatment options, practical aspects of initiating/continuing prophylaxis, dealing with bleeds, and ongoing aspects of treatment, including possible inhibitor development. Over time, optimizing treatment efficacy, in which individualizing therapy around activities can play a role, and long-term considerations, including retaining joint health and tolerance maintenance, become increasingly important. The evolving treatment landscape is creating a need for continually updated guidance. Multidisciplinary teams and peers from patient organizations can help provide relevant information. Easily accessible, multidisciplinary comprehensive care remains a foundation to care. Equipping parents early with the knowledge to facilitate truly informed decision-making will help achieve the best possible longer-term health equity and quality of life for the child and family living with hemophilia.

**Plain language summary:**

**Points to be taken into account to help families make decisions to best care for children born with hemophilia**

Medical advances are providing a range of treatment options for adults and children with hemophilia. There is, however, relatively limited information about managing newborns with the condition. Doctors and nurses can help parents to understand the choices for infants born with hemophilia. We describe the various points doctors and nurses should ideally discuss with families to enable informed decision-making. We focus on infants who require early treatment to prevent spontaneous or traumatic bleeding (prophylaxis), which is recommended to start before 2 years of age. Families with a history of hemophilia may benefit from discussions before pregnancy, including how an affected child would be treated to protect against bleeds. When mothers are pregnant, doctors can explain investigations that can provide information about their unborn child, plan for the birth, and monitor mother and baby to minimize bleed risks at delivery. Testing will confirm whether the baby is affected by hemophilia. Not all infants with hemophilia will be born to families with a history of the condition. Identification of hemophilia for the first time in a family (which is ‘sporadic hemophilia’) occurs in previously undiagnosed infants who have bleeds requiring medical advice and possibly hospital treatment. Before any mothers and babies with hemophilia are discharged from hospital, doctors and nurses will explain to parents how to recognize bleeding and available treatment options can be discussed. Over time, ongoing discussions will help parents to make informed treatment decisions:

• When and how to start, then continue, prophylaxis.

• How to deal with bleeds (reinforcing previous discussions about bleed recognition and treatment) and other ongoing aspects of treatment.

○ For instance, children may develop neutralizing antibodies (inhibitors) to treatment they are receiving, requiring a change to the planned approach.

• Ensuring treatment remains effective as their child grows, considering the varied needs and activities of their child.

## Visual Abstract


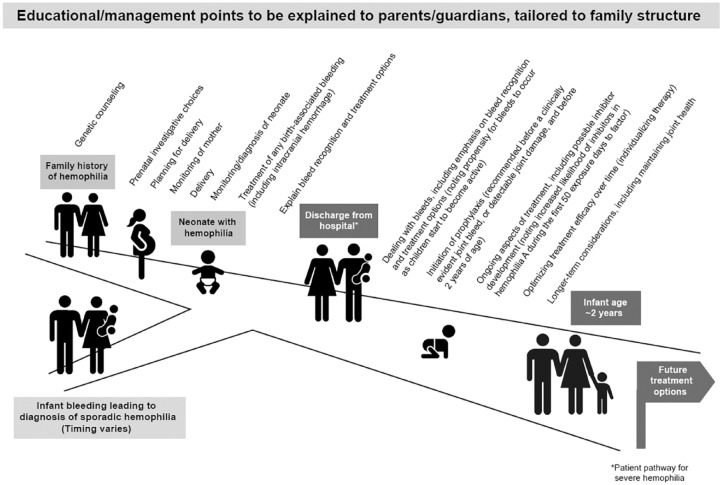


## Introduction

Recent advances in therapeutics are providing a wide range of treatment options for adults and children with hemophilia. Although therapeutic choices are also increasing for the youngest children with severe disease, challenges remain about early management decisions, as supporting data are currently more limited. Parents and healthcare professionals are tasked with helping children achieve an inclusive quality of life and maintain good joint health into adulthood. Early treatment decisions and optimizing medium-term health outcomes will position individuals born today in the best possible health to realize the full advantage of future definitive interventions (e.g. gene therapy) that may be available to them in adulthood.

With the occurrence of sporadic hemophilia as well as hemophilia in individuals with an established family history of the condition, previously untreated patients (PUPs) will continue to present to physicians. Diagnosis as early as possible will contribute toward avoiding critical bleeding. Once patients have been identified, outcomes will be influenced by a range of subsequent management decisions.

This article intends to guide multidisciplinary teams in their discussions with parents to help inform shared decision-making relating to PUPs with hemophilia A or B, including prenatal considerations and hemophilia management during the first years of life. It will cover individuals who require primary prophylaxis, definitions of which vary slightly in terms of timing,^1^ but we would recommend initiating this before a clinically evident joint bleed,^2^ or detectable joint damage,^3^ and before 2 years of age.^2^ Available evidence will be presented with reference to existing guidelines, such as those provided by the World Federation of Hemophilia (WFH),^4^ and in the context of published and emerging data, highlighting areas of debate. Fundamentally, parents should be well informed and the multidisciplinary clinical team members have a key role in facilitating this, as well as directing parents toward complementary information produced by patient organizations.

## Overview of management

**Figure 1.**
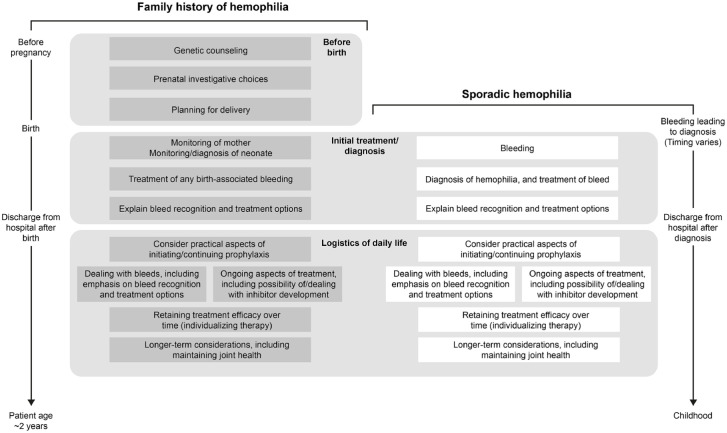
Plan to guide the management of previously untreated patients with hemophilia A or B who will require prophylaxis.

Key points to help guide the management of PUPs with hemophilia are shown in Figure 1 and described in further detail below. Based on available evidence, making timely decisions will improve outcomes.^5–7^

At the outset, it should be appreciated that opportunities and options for families affected by hemophilia vary between countries. In developing regions, a range of challenges, including low awareness of the condition, issues with healthcare infrastructure, and quality-assured diagnostics, as well as lack of factor concentrates, may affect management.^8^ The information in this article should be interpreted/applied as applicable to the country in which families are being counseled.

## Key issues to be considered pertaining to pregnancy and birth

### Before pregnancy

For individuals with a known family history of hemophilia, discussions should begin prior to pregnancy. This will help known or potential carrier women and their partners to understand the implications of hemophilia and proactively prepare for the possibility of having a child with the condition.^9^ Future mothers should have access to multidisciplinary care, highlighting the importance of contact with specialized hemophilia treatment centers and offered combined obstetric/hemophilia clinic appointments, where available. Genetic counseling, including information about reproductive implications and choices, should also be offered.^4,9^ Choices for reproductive investigations may be explained, with reference to the possibility of *in vitro* fertilization with preimplantation genetic diagnosis, where appropriate, legally possible, and desired by families. Alternatively, for other carriers who become pregnant, prenatal testing and sex determination may subsequently be performed, as described below. Consideration should be given to including/accommodating partners, as appropriate.

### From the first trimester

Available prenatal investigative choices vary by country and region, and include a range of possible options.^10–12^ For example, testing of free fetal DNA in the maternal blood (which can be performed around weeks 9–10 of pregnancy), chorionic villus sampling (weeks 10–13), ultrasound (from week 11), and amniocentesis (weeks 15–22) can all be considered. Fetal cord blood sampling (cordocentesis), another possible prenatal investigative option that may confer various risks,^13^ is rarely used. In developing countries where there are no tests of this type, counseling and coagulation screening, for the mother’s pregnancy planning, should be offered^12^ to minimize her risk of bleeding. All potential carriers should at least be informed about the possibility of giving birth to a boy with hemophilia and how to recognize or suspect the disease in their children after birth. Possible bleeding risks associated with any invasive procedure (e.g. fetal scalp monitoring to use of ventouse or forceps) should be considered, together with any necessary prophylactic cover for the mother.

When fetuses are found to be female, invasive prenatal testing, available to diagnose hemophilia in males, would normally be avoided. However, the possibility of skewed lyonization and resultant reduced factor levels into the hemophilia range^14^ should be borne in mind and parents counseled. A cord blood sample is advisable, when possible, to identify female neonates with reduced factor levels to then proceed to cranial ultrasound, as for boys born with hemophilia.

### Birth-related points

Ongoing discussions will help guide planning of the clinical management of pregnant carriers and their unborn children prior to, and during, birth. With close cooperation between the obstetric and hemophilia teams (ideally within a dedicated hemophilia treatment center) during pregnancy, carriers’ factor VIII (FVIII)/factor IX (FIX) levels should be regularly assayed to help determine the risk of bleeding during delivery and postpartum.^4^ Although FVIII levels in carriers increase during pregnancy,^15^ achieving only low ‘normal range’ FVIII levels in the third trimester is arguably not ‘normal’ for the final stages of pregnancy. As such, childbirth can still result in mothers experiencing abnormal bleeding. In addition, levels of both FVIII and von Willebrand factor (VWF) can decrease rapidly after birth and full bleeding history should be sought.^16,17^ Levels of FIX do not show marked increases during pregnancy.^15^

As part of advanced planning, written delivery plans developed in consultation with the mother with/without her partner will encompass a range of items.^4,10,11,18,19^ These include plans relating to the mother’s chosen hospital and mode of delivery (which, whether vaginal or cesarean, should be atraumatic and avoid invasive fetal monitoring), plans for, or contraindications to, regional anesthesia, and possible hemostatic treatment to cover delivery and the mother’s postpartum period, including the type and availability of factor therapy. Consideration of thromboprophylaxis [mechanical (e.g. graduated compression stockings/intermittent calf compression stockings) and/or anticoagulant treatment] will depend on individualized assessment of bleeding risk and the mode of delivery. A mother should have a supply of any necessary factor concentrate at home in case of unexpected complications, such as premature or precipitous labor resulting in her being taken to a more local, nonhemophilia specialist hospital in an emergency.

Bleeds in neonates can arise as a consequence of trauma during labor and delivery;^10^ it has been reported that up to 3–4% of male infants with hemophilia experience some cranial bleeding during this period.^20,21^ Intracranial hemorrhage (ICH) is a severe complication, and a recent systematic review and meta-analysis showed a pooled cumulative incidence of 2.1 (95% confidence interval: 1.5–2.8) per 100 hemophilia live births.^22^ Data from an earlier PedNet study involving 926 neonates showed vaginal delivery and cesarean section to have similar risks for ICH, with incidences of 2.4% and 1.7%, respectively, in newborns with hemophilia,^23^ although evidence in this regard has been conflicting.^11^ Overall, ICH in hemophilia has a mortality rate of approximately 20%^24^ and at least one-third of surviving neonates experience severe sequelae.^10^

### After delivery and during the neonatal period

It is important to monitor carrier mothers for early or delayed postpartum hemorrhage, and hemostatic measures should be employed as needed.^4^

For children newly born to carriers, a range of points should be considered.^4,10,11,18,19,25^ In the absence of technical assay/sampling problems, cord blood testing will likely confirm or exclude a diagnosis of hemophilia in the first few hours after birth, thereby further aiding future management. However, given the possibility of contamination of cord blood by the blood from the mother, if FVIII or FIX levels as measured via cord blood are not within the expected hemophilia range of the family index case, it may be appropriate to perform repeat testing, sampling a peripheral vein, to confidently exclude hemophilia before the child is discharged from hospital. Although most cases of hemophilia A can be diagnosed at birth, FVIII levels can vary slightly at this time, rising transiently into the low normal range because of the acute-phase stress response at birth; therefore, repeat testing at around 6 months of age may be required.^25^ This will identify true baselines of individuals with potential mild hemophilia A. Repeat testing may also be required to identify individuals with mild hemophilia B, as FIX levels are significantly reduced at birth, particularly in preterm infants, which may confound diagnosis of mild, but not severe or moderate, disease.^25^ The importance of prompt diagnosis of skewed FVIII or FIX levels in female offspring at risk of having hemophilia (i.e. obligate carriers who have fathers with known hemophilia, or potential carrier daughters of carrier mothers) is being increasingly recognized,^26^ with new nomenclature proposed to aid both diagnosis and management.^27^ Skewed lyonization^14^ results in reduced factor levels in affected females, most commonly into the mild hemophilia range,^28^ but with a small minority into the moderate or even severe factor level category. Although this is not a current widespread practice, a cord blood sample in a female neonate should be recommended, to give early opportunity to identify significant skewed lyonization and resultant low levels, or to reassure parents of factor normality.

Where prenatal testing was not performed or not available, boys born to known or potential carriers should be presumed to be affected by hemophilia unless confirmed otherwise.^11^ Plans pertaining to heel pricks, routine vaccinations, and administration of vitamin K should also be considered and discussed with parents before birth. For vitamin K, oral delivery offers the safest administrative option but, in any given healthcare system/institution, it is important to confirm who will be responsible for ensuring the course of multiple doses is completed. It may be appropriate to refrain from intramuscular injections or heel pricks until results from coagulation testing are available.^10^ Without delaying the management of the neonates, the general approach should be to use a peripheral vein for all sampling instead of heel prick with attention to adequate pressure, until low factor levels have been excluded or confirmed. However, the prerequisite for this will be a staff/nurse experienced in venipuncture of the neonates. The possibility of bleeding should be borne in mind, with newborns receiving a thorough physical examination and specialist treatment as necessary in the days and weeks after birth. Preterm neonates with hemophilia should be managed on an individual basis, with delivery (when possible) and ongoing care taking place in hemophilia treatment centers in collaboration with relevant services (obstetric, neonatology, etc).^11^

Although this may vary by geographical region, data have shown the most common hemorrhagic complications in neonates include circumcision site bleeding and head bleeds.^29^ Bleeding in neonates with hemophilia exhibits a different pattern from that in older children with the condition.^10,25,30^ Bleeding rarely occurs in muscles and joints, but can present as iatrogenic bleeding, including oozing/excessive hematoma after intramuscular vitamin K administration, heel prick sampling, or venipuncture, as well as postdelivery cephalohematomas, extracranial hemorrhages, or ICH.^10,11^ ICH symptoms can be subtle and nonspecific, occurring more frequently in children with severe bleeding disorders during the first 2 years of life and in children not receiving prophylaxis. In children, ICH often occurs without documented trauma, and incidences of 6.4 and 4.2 per 1000 patient years have been reported for hemophilia A and B, respectively.^31^ Neuroimaging modalities to diagnose such bleeding include ultrasound, computed tomography scans, and magnetic resonance imaging (MRI), but not conventional radiography.^11^ Notably, the focus must be placed on ultrasound for initial diagnosis; this is effective, less demanding for a newborn or infant than an MRI, and more likely to result in earlier treatment. Cranial ultrasonography should be recommended as part of birth planning. Diagnostic imaging is particularly important for preterm infants, after difficult deliveries, for neonates who exhibit facial bruising, or when extracranial bleeding is apparent.^11^

Around one-third of cases of hemophilia occur in families with no history of the condition,^30^ arising as a consequence of a genetic mutation that had not been previously recognized within a family. In such individuals, the initial presentation may be bleeding in the neonatal period,^25^ acting as a diagnostic indicator for this ‘sporadic hemophilia’. For parents of a child affected by sporadic hemophilia, the suddenness of diagnosis is likely to compress the timelines for discussion of/education about the condition, compared with situations in which there is a family history of the disease.

As not all bleeds in the neonatal period are apparent around the time of delivery,^11^ prior to discharge from hospital, parents of affected neonates should be coached about signs or symptoms to look out for that might indicate a bleed,^25^ for example, inconsolable crying, poor feeding, distress on moving a particular limb/joint. Parents should have clear instructions and contact details for emergency and out-of-hours advice,^32^ to enable prompt access to assessment and treatment. Discussions should have taken place to decide upon the choice of factor concentrate to be used for bleed management, and consideration given to providing parents with an appropriately small vial (e.g. 250 IU) of the chosen factor to be taken to a clinic in the event of any emergency attendance (depending on local practice, etc). Particularly for out-of-hours care, to expedite timely assessment and treatment if/when necessary, emergency contact pathways to the specialist hemophilia team should be confirmed. Immediate intervention may not be necessary for mild bruising. Other bleeds require prompt appraisal, dosing calculation, and management.^11^

## Bleed recognition in infants

Bleed recognition in neonates and infants differs from that in older children. Data from infants with hemophilia during the first 2 years of life have shown that, beyond the neonatal period, common complications include soft tissue/intramuscular hematomas, oral/nasal bleeding, head injuries, and joint bleeds, with joint disease then becoming a characteristic of older age groups.^33^ In relation to this, there may be a propensity for bleeds to occur as children start to become active (crawling as well as pulling up to stand and cruise).

For patients of all ages, joint bleeds can result in a decreased range of motion or cause difficulties for joint use.^3,4^ Joint bleeds in infants may manifest with affected individuals avoiding mobilization, adopting protective postures, or exhibiting different patterns of movement/limping. Less specific symptoms of apparent distress (e.g. inconsolable crying) may also be evident.

Any head injury should receive immediate attention because of the possibility of ICH, which, in infants, may be suggested by somnolence or feeding difficulties. In some instances, birth-related ICH may only be apparent after initial hospital discharge (not resulting from any further trauma) and parents should be advised as to the signs of this and the importance of seeking help as soon as possible.

Oral/nasal bleeding should be visibly apparent, although small children can swallow a lot of blood. Symptoms of soft tissue bleeds vary according to the site.^4^ Muscle bleeds can present diagnostic difficulties, but pain, swelling, and/or loss of movement may be evident.^3^ Renal hemorrhage can manifest as swelling in the abdomen and/or hematuria,^4^ while hematemesis, melena, or fresh blood loss in or with the stool (hematochezia) are indicative of gastrointestinal bleeding.^34^

It should be emphasized that if there are any doubts about a hemorrhagic complication, an assessment should be carried out immediately, even if out-of-hours. A hemophilia specialist should also be consulted in the event of complications not related to hemophilia, such as diarrhea or fever, to prevent iatrogenic complications in both older and younger patients.

## Key issues to be considered for subsequent management of neonates and infants with hemophilia A or B

### Potential treatment options

#### Practicalities of different options and the scientific evidence supporting their use

Parents should be fully informed of the various options available for preventing and managing bleeds in their children, with reference to current guidelines^4,35^ and existing data. For those with a family history of hemophilia, these conversations should have started in the prenatal and pregnancy stages. Desmopressin is contraindicated in patients aged <2 years because of the risk of hyponatremia,^4^ and there is limited evidence for use of systemic antifibrinolytic therapy in newborns and infants. As hemophilia can be managed with replacement of missing clotting factor, in some parts of the world, cryoprecipitate or fresh frozen plasma may be the only option available to achieve this, although these are not recommended because of concerns about virus transmission.^4^

**Figure 2.**
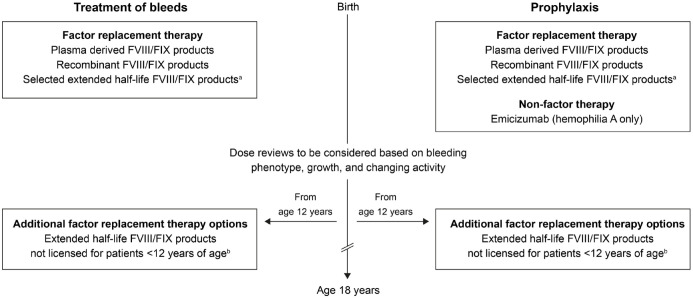
Treatment options for previously untreated patients (PUPs) with hemophilia A or B who will require prophylaxis. Studies of extended half-life products conducted in PUPs and other pediatric patients are included in Supplemental Table 1. Product availability will vary between countries. Licensing differs between the European Union and the United States. FVIII, factor VIII; FIX, factor IX; rFVIIIFc, recombinant factor VIII Fc fusion protein; rFIXFc, recombinant factor IX Fc fusion protein; rIX-FP, recombinant factor IX albumin fusion protein. ^a^rFVIIIFc,^36,37^ rFIXFc,^38,39^ and rIX-FP^40,41^ are approved for all age groups in the European Union and the United States; in the United States, rurioctocog alfa pegol,^42^ turoctocog alfa pegol,^43^ and nonacog beta pegol^44^ are also approved for children. ^b^In the European Union, use of rurioctocog alfa pegol,^45^ turoctocog alfa pegol,^46^ damoctocog alfa pegol,^47^ and nonacog beta pegol^48^ is licensed only in patients ⩾12 years of age; in the United States, damoctocog alfa pegol^49^ is licensed only in patients ⩾12 years of age; in both the European Union and the United States, damoctocog alfa pegol^47,49^ is not licensed for PUPs.

Clotting factor concentrates have been the treatment of choice for people with hemophilia, but in recent years, nonfactor therapy has also become available.^4^ These options^36–49^ (summarized in Supplemental Table 1 and Figure 2) are considered in detail below, including in relation to practicality of use and the scientific evidence available in relation to treatment of PUPs.

#### Factor replacement therapy

A range of clotting factor concentrates are available to facilitate factor replacement therapy in patients with hemophilia,^50^ and principles to help guide selection have been published by the WFH.^51^ Plasma-derived, recombinant standard, and extended half-life recombinant products are available; therapeutic choice should be guided by evidence-based medicine and local criteria including availability, cost, and patient preference.^4^ Clotting factor products require intravenous administration, and venous access is a particularly important consideration when treating small children with hemophilia.^52^ Indeed, data have shown that with prophylaxis administered with at least three infusions of clotting factor per week in patients with hemophilia A under 3 years of age, central venous access devices have been used in 34–88% of cases, depending on the regimen and cohort.^53^ However, these data were based on therapies with standard half-lives. Recombinant factor products with extended half-lives have been developed to give the option of reduced treatment burden by requiring less frequent administration than standard half-life therapies or to increase the levels of protection if maintaining the dosing frequency; these rely on PEGylation or Fc fusion technology for hemophilia A.^54^ For hemophilia B, in addition to these, fusion technology linking FIX with recombinant albumin has also been used.^55^ Polyethylene glycol (PEG) has been used to prolong the half-life of other drugs, but with the possibility of products for the treatment of hemophilia being used lifelong from infancy and a lack of safety data relating to this, PEGylated coagulation factors are not universally approved by regulatory agencies for patients <12 years of age; no such products are licensed for these younger patients in Europe but some are available in the United States. In contrast, fusion products are not subject to this age restriction.

#### Nonfactor therapy

As with any treatment, replacement factor therapy has limitations. While modified factor molecules have resulted in products with extended half-lives compared with conventional standard half-life factor therapy, these still require intravenous administration and, as considered below, their efficacy may be compromised by inhibitor formation or poor compliance; consequently, nonfactor replacement therapy has been investigated.

Emicizumab, a recombinant, humanized, bispecific monoclonal antibody, is the only nonfactor therapy currently approved for the treatment of hemophilia A. This mimics the function of missing activated FVIII in coagulation by bridging (activated) FIX and factor X (FX), to enable activation of FX.^56^ Subcutaneous administration of emicizumab can provide effective prophylaxis in patients with hemophilia A and inhibitors.^57^ It is also approved in many countries for prophylaxis in patients with hemophilia A who do not have inhibitors (but, in the European Union, this is currently limited to patients without inhibitors who have severe hemophilia A).^58,59^ It is easier to train parents to administer subcutaneous than intravenous prophylaxis, affording earlier independence from the hospital team, although parents will necessarily be dependent on hospital staff for emergency intravenous administration of FVIII concentrate in the event of trauma of sufficient concern to require additional factor treatment. Although, where licensed, emicizumab is available for all age groups, more data are required for guidance in the very youngest patients. A number of other issues remain to be resolved.^60^ Therefore, approaches for initiating prophylaxis and use of therapeutic options in very young children will differ, although more data will be provided from an ongoing clinical study involving emicizumab.^61^

It remains to be determined whether naturally reduced levels of FIX and FX in neonates compromise the efficacy of emicizumab, and whether emicizumab treatment affords protection against ICH. In addition, given poor compliance with inhibitor screening guidelines in patients with nonsevere hemophilia A,^62^ there are concerns about inhibitor screening with on-demand factor concentrate use in patients also treated with emicizumab. Based on extrapolation from factor exposure modeling in patients with mild hemophilia A,^63^ the first 20 exposure days to FVIII concentrate may be spread over many years. Given the potential for inhibitor development to occur relatively early in the overall time course of exposure to factor concentrate (see below), there is the potential for undiagnosed inhibitors to emerge in early years of life, possibly compromising treatment of acute bleeds. Consequently, members of the multidisciplinary team managing patients treated with emicizumab should be attentive to inhibitor screening at correct times after exposure to factor concentrates, ensuring this is performed with correct laboratory reagents.^64^ Establishing/maintaining tolerance to FVIII therapy will remain important. Beyond direct effects on coagulation, any long-term effects of emicizumab in comparison to potential benefits provided by FVIII also remain to be clarified; for example, the role of FVIII in maintaining bone health is currently under consideration,^65^ although such studies are speculative.

#### Possible future treatment options

A range of other treatments are under investigation, and it may be of value to briefly mention these to parents during early counseling. For example, BIVV001^66,67^ (efanesoctocog alfa, Sobi and Sanofi) is an investigational factor-based therapy in which fusion of a VWF domain and two XTEN polypeptides to recombinant FVIII Fc fusion protein (rFVIIIFc) provides FVIII stabilization and steric shielding resulting in an FVIII molecule with a half-life longer than existing FVIII products and higher levels of protection, with once weekly dosing. A phase III study in pediatric previously treated patients is currently ongoing.^68^

Other nonfactor molecules intended to provide prophylaxis via subcutaneous delivery are also in clinical trials: a second-generation bispecific monoclonal antibody (Mim8; Novo Nordisk);^69^ monoclonal antibodies targeting tissue factor pathway inhibitors to increase the potential for thrombin generation by ensuring that activated FX and the activated factor VII–tissue factor complex remain active^70,71^ (e.g. concizumab; Novo Nordisk,^72,73^ marstacimab; Pfizer^74,75^); and fitusiran (Sanofi^76,77^), a small interfering RNA molecule that decreases production of antithrombin by blocking translation of the antithrombin-encoding *SERPINC1* messenger RNA.^78^ In addition, preclinical data support the process of targeting activated protein C, another natural inhibitor of coagulation.^79,80^ Such investigational rebalancing technologies are unlikely to be available to PUPs in the near future and more detailed consideration is beyond the scope of the current article but can be found elsewhere.^81,82^

Gene therapy has the ultimate goal of achieving phenotypic cure; positive results have been reported in patients with hemophilia^83–86^ and a number of approaches are in various stages of clinical development (phases I–III). The first gene therapy for hemophilia A (valoctocogene roxaparvovec; Biomarin) has recently been licensed for adults by the European Medicines Agency,^87^ with the first gene therapy for adults with hemophilia B (etranacogene dezaparvovec; CSL Behring) subsequently approved by the U.S. Food and Drug Administration.^88^ The impact of tolerance toward the deficient protein (FVIII/FIX) on eligibility for gene therapy remains to be determined. Material providing more information on this technology has recently been published,^89^ but it is not currently an option for children.

### Practical aspects of prophylaxis

#### Patient eligibility

The benefits of prophylaxis in patients with severe hemophilia have been clearly demonstrated,^7,90^ and the WFH recommends this approach be used to prevent bleeds.^4^ Discussions with parents should explain the rationale for prophylaxis. Parents should be helped to understand the ways in which prophylaxis improves the lives of patients and their families, and that, given variation in bleeding phenotype, these benefits are not necessarily limited to cases of severe disease. While prophylaxis with factor replacement improves trough levels of FVIII/FIX, there is ongoing debate with regard to optimum values, but maintaining these above 3 IU/dl or higher has been advocated,^91^ with prophylaxis now likely to include individuals with moderate disease, particularly if associated with a bleeding phenotype of concern. For patients with hemophilia A, primary prophylaxis via subcutaneous injection of emicizumab^58^ will also be an option, where licensed.

From a practical point of view, the various therapeutic choices should be discussed with parents with reference to the available products and latest data, venous access for factor replacement therapy in small children, and the timepoint at which prophylaxis is initiated (as considered further below). Parents should be helped to understand how the benefits of prophylaxis outweigh the burden – home-based treatment and self-infusion in older patients can subsequently reduce the therapeutic burden.

#### Timing of prophylaxis, therapeutic choices, and dealing with breakthrough bleeds

With recommendations to start primary prophylaxis before or after a first joint bleed, the PedNet Hemophilia Registry protocol states that, in practice, factor-based prophylaxis has generally been started between 1 and 2 years of age.^92^ Decisions about providing therapy are made on an individual basis^11^ and individualized prophylaxis, with treatment tailored to bleeding phenotype and desired level of protection, is advocated by the WFH.^4^

Clinical trial and real-world data providing evidence of the efficacy and safety of newer treatments continue to become available to help inform decision-making. For instance, recent data from a clinical study of rFVIIIFc, involving 103 previously untreated pediatric patients with severe hemophilia A, 80 of whom were aged <1 year, reported an overall median annualized bleeding rate (ABR) of 1.49 [interquartile range (IQR) = 0.00–4.40].^93^ For hemophilia B, in a clinical study using nonacog beta pegol to treat 37 patients aged <6 years (median age = 1.0 years) who were previously untreated or had <3 exposure days to FIX-containing products, the modeled mean ABR for the 28 patients receiving weekly prophylaxis was 0.31.^94^ There is, however, relatively little data on PUPs compared with older patients, and it is important to encourage data collection from PUPs/younger age groups to ensure these patients can benefit from the evolving treatment landscape, including nonreplacement therapy. Real-world evidence from the PedNet cohort involving 141 previously treated pediatric patients with hemophilia A, 28 of whom were aged <2 years and 79 had inhibitors, showed 58% of patients to have no bleeds during a median of 9.8 (IQR = 3.6–19.8) months of treatment with emicizumab; most patients who did experience bleeds (42 of 58) had inhibitors.^95^

Parents should understand that, regardless of the choice of regimen, prophylaxis reduces, but does not completely abolish, the risk of bleeding. As previously mentioned, the importance of recognizing bleeds in both neonates and infants should be explained to parents, as should the importance of treatment individualization based on activity and lifestyle as the child grows up (see below). This should help them to optimize the outcome for their child, identifying breakthrough bleeds and thereby facilitating timely intervention. Additional information that physicians may provide to families in the context of dealing with breakthrough bleeds could include discussion about the possibility of increasing the intensity of prophylaxis and the potential for inhibitor development (see below). Families should be educated to understand that prophylactic choices afforded by therapies with different mechanisms of action and routes of administration provide the potential to switch between therapies to suit prevailing circumstances.

### Safety aspects, including inhibitors

#### Summary of safety profiles of available options

When compared with well-characterized therapies, there is a requirement for more information in relation to the adverse events associated with new treatment options. In this context, long-term follow-up studies/real-world data are required to fully assess safety.

#### Establishing tolerance, initial failure of tolerance, and immune tolerance induction

Inhibitor development is a serious complication of factor replacement therapy, affecting one-third of PUPs with severe hemophilia A, mostly during the first 50 exposure days.^96^ The frequency of inhibitor occurrence in patients with hemophilia B is less well defined, but recent data involving an unselected cohort of patients with severe hemophilia B from the PedNet cohort have reported a cumulative inhibitor incidence of about 10% at 75 exposure days.^97^

As previously mentioned, parents should be made aware of the possibility of the development of factor inhibitors, including mention of risk factors and when to suspect their occurrence, explaining how the hemostatic effects of factor therapy will be compromised. Tolerance to factor therapy is important for achieving hemostasis in potential emergency bleed/trauma scenarios and also in relation to possible surgery. Risk factors for inhibitors include race, family history, genetic profile, and hemophilia severity, as well as the intensity and type of replacement product used, although there is debate and controversy around the latter.^4^ The chance to influence even environmental risk factors is extremely limited. Detailed discussion of risk factors for inhibitors is beyond the scope of this article, but it should be noted that the immune response to FVIII/FIX products is poorly understood and there is an absence of sufficient/complete evidence around this topic,^4^ confounding reliable prediction of individuals in whom inhibitors will develop. From a practical point of view, inhibitors are extremely relevant, but so is ensuring patients can receive prophylaxis and parents should be informed of the efficacy of the relevant options.

The implications of the changing hemophilia landscape for managing inhibitors have been discussed in updated guidance.^98^ Product options for managing bleeds in patients with factor inhibitors have been summarized in WFH guidelines.^4^ Replacement factor therapy may be used for low-responding inhibitors, whereas patients with high-responding inhibitors may require bypassing agents (recombinant activated factor VII or activated prothrombin complex concentrate, with caveats for those receiving emicizumab prophylaxis) for breakthrough bleeds and trauma or to cover surgical procedures.

Beyond the treatment of acute bleeds, immune tolerance induction (ITI) can eliminate inhibitors via repeated administration of factor.^99^ Detailed discussion of ITI is beyond the scope of this article, but with the ongoing requirement for factor therapy or bypassing agents for dealing with bleeds, and given the possibility of inhibitors compromising the effectiveness of such treatment, tolerance is important to ensure the efficacy of future interventions and protect against irreversible damage. Evidence-based guidelines support early intervention to successfully eradicate inhibitors.^100^ Considering the changing therapeutic landscape in hemophilia A, less demanding ITI approaches, with or without emicizumab for optimal bleed protection, are being investigated,^101,102^ and updated consensus recommendations have recently been published.^103,104^ For example, the ‘Atlanta protocol’ describes ITI in pediatric patients with hemophilia A and inhibitors receiving concomitant emicizumab prophylaxis,^101^ while revised ITI guidance from the UK Haemophilia Centre Doctors’ Organisation (UKHCDO) recommends using emicizumab prophylaxis to reduce bleeding while enabling low-dose and reduced-frequency factor therapy in most children receiving ITI.^104^

Currently, there is no evidence that nonfactor-based prophylaxis offers any advantage in reducing inhibitor occurrence, although an ongoing study is investigating whether the context of concurrent FVIII exposure may impact inhibitor rates.^105^ The agent used to start prophylaxis is a key management decision and, as with factor therapy, the issue of timely inhibitor detection will be crucial. Although rare, it is also necessary to be aware of the possibility of the development of antidrug antibodies (ADAs) to emicizumab, which could impact the pharmacokinetics/pharmacodynamics of the agent and affect its efficacy. In the HAVEN clinical trial series (HAVEN 1–4), 3 of 398 patients (0.75% of the overall clinical trial population) developed ADAs with neutralizing potential, with 1 patient discontinuing emicizumab treatment because of loss of efficacy.^106^ In the subsequent STASEY trial, 10 of 193 patients (5.2%) developed ADAs, none of which affected efficacy,^107^ while postauthorization data found 1 case of emicizumab-neutralizing ADAs in a 6-year-old boy with severe hemophilia A and inhibitors.^108^ Collation of registry data is crucial.

All monoclonal antibodies may be impacted by ADAs, which cannot be assumed to be similar either within or across classes. ADA development with Mim8 is being assessed.^109^ For antibodies targeting tissue factor pathway inhibitors, with concizumab, for instance, 25% of patients in the explorer4 (recruiting patients with hemophilia A or B and inhibitors)/explorer5 (recruiting patients with severe hemophilia A without inhibitors) trials developed ADAs during the main and extension phases, with no apparent clinical effect, with the exception of one patient for whom the clinical impact was inconclusive.^110^

It is important to consider potential antibody-neutralizing effects for all treatment options – both those that are currently available and those that may become available in the future – distinguishing between the value of tolerance to prophylactic agents and tolerance to drugs required to treat acute bleeding.

### Individualized therapy

#### Optimizing treatment efficacy

Whereas nonfactor prophylaxis tends to be administered with fixed dosing at specific intervals, with factor therapy, prophylactic dosing (product amount and interval between treatment) can be adjusted based on patient response. With factor therapy, tailored prophylaxis regimens can help to optimize treatment efficacy,^4^ and pharmacokinetic analyses have shown the value of modifying dosing in pediatric patients.^111^ Maintaining minimum trough levels is particularly important in children, and factor products with an extended half-life may enable higher trough levels to be achieved over an extended period.^112^ Monitoring and timing of peaks can also be considered when optimizing individualized treatment plans,^113,114^ although evidence for this remains limited. Pharmacokinetic-guided prophylaxis^115^ has shown greater area under the pharmacokinetic curve (which reflects greater exposure of the patient to the product), and higher trough levels provide increased bleed protection. As young children grow up, attending primary and then secondary school, adapting therapy to their lifestyle, as they and their peers become stronger and faster, increasing their physical activities with resultant greater risk of injury/impact in sport, timing peaks to coincide with higher levels of physical activity will provide greater bleed protection. Indeed, patient-centric outcomes advocate targeted approaches relating to activity levels;^116,117^ the incidence of a bleed occurring in physically active children with hemophilia has been shown to decrease by 2% for every 1% increase in the trough level (modeling factor levels based on factor treatment of children aged between 4 and 18 years of age who had moderate or severe hemophilia).^118^

#### Retaining treatment efficacy over time

A range of items should be considered to help retain treatment efficacy over time. These include changing situations as patients grow up, their activity levels, adherence to prescribed prophylaxis, as well as the patient’s general health and any other conditions that may present. Although adherence may be high when parents are responsible for administering treatment to their child, there may be a decline when adolescents assume this responsibility. However, the stereotype of declining adherence in adolescent chronic disease management may be less apparent in hemophilia with the availability of wraparound comprehensive care.^119^ If adherence or treatment burden/efficacy issues do arise, there is now the possibility of switching between therapies,^120^ which may allow patients to benefit from the different advantages provided by various products at different times of their lives, helping patients to optimize, for example, treatment convenience and treatment efficacy. There is currently no evidence to suggest that switching between different factor products significantly influences inhibitor development;^121^ however, it is not known whether tolerance is maintained if patients change to nonfactor therapy, particularly if they have received successful ITI earlier in life.

### Logistics of daily life

#### Shared decisions on treatment, support, and reevaluation

Healthcare providers should explain the aforementioned points to parents to help inform decision-making, giving them a short- and medium-term view for their child, including logistics for daily life and the treatment journey. Ultimately, allowing children to access routine childcare can provide stimulation and avoid the risk of overprotection;^52^ where possible, parents should be equipped to provide information about hemophilia to others entrusted with the care of their child. Comprehensive care teams often provide additional information and care plans to daycare/nursery/kindergarten/school staff; this ensures that staff members are sufficiently confident to know when to call for help in a timely manner, as well as facilitating inclusion rather than exclusion of children with hemophilia in physical activities with their peers.

#### Long-term considerations

As children grow up, their role in decision-making will evolve. They should become more involved in both preparation and then administration of their prophylaxis during their primary school years, as well as then joining the clinical conversation about the prophylaxis that best suits them. Including children in ongoing discussions from an early age is key to ensuring that they appreciate the principles of care. Given the subtle development of arthropathy and limitations of early detection,^122^ maintaining joint health should be a focus during childhood. Point-of-care ultrasound (POCUS)^123^ and scheduled physiotherapy for musculoskeletal reviews [e.g. Hemophilia Joint Health Score (HJHS)] provide an important opportunity to reinforce messages about safe inclusion in activities and sport with their peers. More detailed imaging (e.g. MRI) may be informative when indicated. Paradoxically, the better the early prophylaxis provided to young children, the less they require interactions with the hemophilia team, and thus, there may be fewer opportunities to reinforce messages about prophylaxis, lifestyle decisions, trauma treatment requirements, and emergency pathways of communication and care.

As hemophilia is a lifelong disorder, there is a continuous process of change in its management as individuals progress through life stages;^124^ while in the last decade, medical advances have resulted in rapidly changing therapeutic options.^125^ Beyond the immediate concerns for managing PUPs, long-term issues that should be considered include maintaining joint health, paying attention to musculoskeletal status, empowering physical activity, and including patients (as individuals or with peers) in activities that are deemed within the protective remit of their chosen prophylaxis (see recent WFH guidance about collision sport^4^). Avoiding spontaneous bleeds, while minimizing the impact of trauma-related bleeding, should help to minimize musculoskeletal damage, with the ultimate goal of avoiding damage that might result in chronic pain. If such damage does occur, adequate physical therapy, pain management, and timely orthopedic interventions should be accessible to minimize the life impact of these changes. Appropriate clinical and laboratory follow-up has a role here. Other possible considerations include minimizing the psychosocial burden of the disease for both the individual and family, which will have a key influence with regard to maintaining quality of life, and may require a greater level of consideration for parents of children with sporadic hemophilia than those with family history of the condition; chronic inhibitor management beyond prophylaxis with nonfactor therapy for those unable to tolerize; and counseling about risk of loss of tolerance if considering switching to nonfactor therapy after successful ITI. The possibilities potentially afforded by future developments may result in PUPs being offered curative gene therapy in their lifetime; they will want to have achieved the best possible outcomes up to this point.

Ideally, the overall aim of treatment should be to achieve a quality of life comparable to individuals without hemophilia. An ultimate goal should be to achieve ‘health equity’.^126^ The options that may be available to patients currently being born with hemophilia may help to achieve this.

## Concluding points

To optimize outcomes, a range of topics need to be discussed with parents to help aid understanding of the early decisions they can make that affect the management of their child/children born with hemophilia (Table 1). For physicians who may not be familiar with the principles of shared decision-making for those affected by hemophilia, the process has been described in detail elsewhere,^127^ while resources that can help with parental understanding have been produced by organizations such as the WFH,^128^ the European Haemophilia Consortium,^129^ and the National Hemophilia Foundation.^130^ The pathophysiology of the disease needs to be considered as do the consequences of bleeding, together with the benefits and risks associated with the different options available for bleed prevention and management. Multidisciplinary teams and peers from patient organizations can help to provide relevant information. Easily accessible care, support, and information will help encourage adherence to prescribed prophylaxis and benefit outcomes.^131^

**Table 1.** Key points to be covered when counseling families who have a child/children born with hemophilia.

Subject area	Points to be covered
Pregnancy and birth (for those with a family history of hemophilia)	• Genetic counseling. • Reproductive implications, choices, and investigations. • Birth planning. • Monitoring of mother and child. • Diagnosis and treatment of neonate.
Bleed recognition	• Bleeds and their complications. • The importance of prompt access to care, including out-of-hours assessment.
Treatment	• The availability and practical use of different treatment options, with reference to the evidence supporting use in PUPs – Factor replacement therapy – Nonfactor therapy – Possible future options
Prophylaxis	• Patient eligibility (including benefits beyond those with severe hemophilia). • Timing. • Therapeutic choices. • Dealing with breakthrough bleeds.
Safety	• Possibility of inhibitor development and the implications of this. • Immune tolerance induction.
Optimizing and retaining treatment efficacy over time	• Hemostatic cover to take into account growth and development. • Possibilities of switching between therapies and tailored prophylaxis with factor replacement. • Maintaining adherence.
Logistics of daily life	• Providing others with the knowledge to care for children while at daycare/nursery/school etc, to enable appropriate involvement in activities. • Long-term considerations to account for management transitions during the treatment journey. • The importance of maintaining joint health.

PUPs, previously untreated patients.

The evolving treatment landscape is creating a need for continually updated guidance.^32,132–134^ With the products currently available, and those likely to arise in the near future, parents and their children have a greater choice than ever. This is a rapidly evolving field. Healthcare professionals need to keep updated and contribute actively, in collaboration with patient organizations, to help patients and their families be aware of the latest innovations, understand them, and appreciate the benefits and potential risks. It is important to provide parents, and ultimately those individuals living with hemophilia as they become older, with the information required to facilitate truly informed decision-making to achieve the best possible health equity and quality of life.

## Supplemental Material

sj-docx-1-tah-10.1177_20406207231165857 – Supplemental material for Considerations for shared decision management in previously untreated patients with hemophilia A or B Click here for additional data file. Supplemental material, sj-docx-1-tah-10.1177_20406207231165857 for Considerations for shared decision management in previously untreated patients with hemophilia A or B by Jan Astermark, Jan Blatný, Christoph Königs, Cédric Hermans, Victor Jiménez-Yuste and Daniel P. Hart in Therapeutic Advances in Hematology

## Declarations

**Ethics approval and consent to participate:** Not applicable.

**Consent for publication:** Not applicable.

**Availability of data and materials:** Not applicable.
