# Supplementary material for: Considerations for shared decision management in previously untreated patients with hemophilia A or B
Source: Ther Adv Hematol. 2023 Apr 17;14:20406207231165857. doi: 10.1177/20406207231165857 (PMC10126613; doi:10.1177/20406207231165857)
Supplement: sj-docx-1-tah-10.1177_20406207231165857 – Supplemental material for Considerations for shared decision management in previously untreated patients with hemophilia A or B [file sj-docx-1-tah-10.1177_20406207231165857.docx]

**Considerations for shared decision management in previously untreated patients with hemophilia A or B**

Jan Astermark,^a^ Jan Blatný, Christoph Königs, Cédric Hermans, Victor Jiménez-Yuste and
Daniel P. Hart^a^

**Jan Astermark**, Department of Translational Medicine, Lund University, and Department of Hematology, Oncology and Radiation Physics, Skåne University Hospital, Malmö, Sweden

**Jan Blatný,** Department of Pediatric Hematology, University Hospital Brno and Masaryk University Brno, Czech Republic

**Christoph Königs,** Clinical and Molecular Hemostasis, Department of Pediatrics, University Hospital Frankfurt, Goethe University, Frankfurt, Germany

**Cédric Hermans**, Hemostasis and Thrombosis Unit, Division of Hematology, Cliniques Universitaires Saint-Luc, Université catholique de Louvain (UCLouvain), 1200 Brussels, Belgium

**Victor Jiménez Yuste,** Hematology Department, Hospital Universitario La Paz, Autónoma University, Madrid, Madrid, Spain

**Daniel P. Hart,** The Royal London Hospital Haemophilia Centre, Barts and The London School of Medicine, QMUL, London, UK

^a^These authors contributed equally to this work.

**Supplemental Table 1. Products currently available for previously untreated patients with hemophilia.**

| **Product** | **Description** | **Additional information** |
| --- | --- | --- |
| Factor replacement therapy | | |
| Plasma-derived FVIII/FIX products | Virally inactivated replacement products derived from pooled human plasma | Licensed for prophylaxis and treating bleeds in all age groups |
| Recombinant FVIII/FIX products | Genetically engineered products avoiding use of human plasma | Licensed for prophylaxis and treating bleeds in all age groups |
| Extended life recombinant FVIII/FIX products | Use Fc fusion technology (and or PEGylation) to extend half-life, thereby reducing treatment burden by requiring less frequent administration than conventional therapies; for hemophilia B, albumin fusion technology has also been used | Licensed for prophylaxis and treating bleeds; agents produced using fusion technology are licensed for all age groups;^1-6^ PEGylated coagulation factors are not universally approved for patients <12 years of age^a^  Published evidence for use of various agents in PUPs includes:   - rFVIIIFc — PUPS-A-LONG study^7^ - rFIXFc — PUPS B-LONG study^8^ - nonacog beta pegol — paradigm^TM^6 study^9^   Other published evidence involving pediatric patients includes:   - rFVIIIFc — Kids A-LONG^10^ and ASPIRE^11^ studies - rurioctocog alfa pegol — NCT02210091^12^ - damoctocog alfa pegol — PROTECT VIII Kids study^13^ - turoctocog alfa pegol — pathfinder^TM^5 study^14, 15^ - rFIXFc — Kids B-LONG^16^ and B-YOND studies^17^ rIX-FP — PROLONG-9FP study^18^ - nonacog beta pegol — paradigm^TM^5^19^ |

| Non-factor therapy | | |
| --- | --- | --- |
| Emicizumab (hemophilia A only) | A bispecific monoclonal antibody, that mimics the function of missing activated FVIII by bridging (activated) FIX and FX, to enable activation of FX | Indicated for prophylaxis (but not treatment of bleeding episodes) for patients with hemophilia A without inhibitors (limited to patients without inhibitors who have severe hemophilia A in the EU), as well as those with hemophilia A with inhibitors;^20, 21^ availability varies between countries. License includes all age groups, but more data are currently required for young patients, including PUPs |

FVIII, factor VIII; FIX, factor IX; PUP, previously untreated patient, rFVIIIFc, recombinant factor VIII Fc fusion protein, rFIXFc, recombinant factor IX Fc fusion protein; rIX-FP, recombinant factor IX albumin fusion protein.
^a^Further details provided in Figure 2 of manuscript.

**References**

1. European Medicines Agency. Elocta Summary of Product Characteristics., <https://www.ema.europa.eu/en/documents/product-information/elocta-epar-product-information_en.pdf> (2021, accessed November 2022).

2. Bioverativ Therapeutics Inc. Eloctate^®^ Prescribing Information, <https://www.fda.gov/media/88746/download> (2020, accessed November 2022).

3. European Medicines Agency. Alprolix Summary of Product Characteristics., <https://www.ema.europa.eu/en/documents/product-information/alprolix-epar-product-information_en.pdf> (2021, accessed November 2022).

4. Bioverativ Therapeutics Inc. Alprolix® Prescribing Information, <http://products.sanofi.us/Alprolix/alprolix.pdf> (2020, accessed November 2022).

5. European Medicines Agency. Idelvion® Summary of Product Characteristics, <https://www.ema.europa.eu/en/documents/product-information/idelvion-epar-product-information_en.pdf> (2021, accessed November 2022).

6. CSL Behring GmbH. Idelvion® Prescribing Information, <https://www.idelvion.com/prescribing-information> (2021, accessed November 2022).

7. Hermans C, Mancuso ME, Nolan B, et al. Recombinant factor VIII Fc for the treatment of haemophilia A. *Eur J Haematol* 2021; 106: 745-761.

8. Nolan B, Klukowska A, Shapiro A, et al. Final results of PUPs B‐­LONG study: Evaluating safety and efficacy of rFIXFc in previously untreated patients with haemophilia B. *Res Pract Thromb Haemost* 2020; 4(Suppl. 1): 479–480.

9. Chan AK, Alamelu J, Barnes C, et al. Nonacog beta pegol (N9-GP) in hemophilia B: First report on safety and efficacy in previously untreated and minimally treated patients. *Res Pract Thromb Haemost* 2020; 4: 1101-1113.

10. Young G, Mahlangu J, Kulkarni R, et al. Recombinant factor VIII Fc fusion protein for the prevention and treatment of bleeding in children with severe hemophilia A. *J Thromb Haemost* 2015; 13: 967-977.

11. Nolan B, Mahlangu J, Pabinger I, et al. Recombinant factor VIII Fc fusion protein for the treatment of severe haemophilia A: final results from the ASPIRE extension study. *Haemophilia* 2020; 26: 494-502.

12. Mullins ES, Stasyshyn O, Alvarez-Román MT, et al. Extended half-life pegylated, full-length recombinant factor VIII for prophylaxis in children with severe haemophilia A. *Haemophilia* 2017; 23: 238-246.

13. Mancuso ME, Biss T, Fischer K, et al. PROTECT VIII kids extension study: Long-term safety and efficacy of BAY 94-9027 (damoctocog alfa pegol) in children with severe haemophilia A. *Haemophilia* 2021; 27: 434-444.

14. Chowdary P, Carcao M, Holme PA, et al. Fixed doses of N8-GP prophylaxis maintain moderate-to-mild factor VIII levels in the majority of patients with severe hemophilia A. *Res Pract Thromb Haemost* 2019; 3: 542-554.

15. Tosetto A, Neff A, Lentz SR, et al. Turoctocog alfa pegol provides effective management for major and minor surgical procedures in patients across all age groups with severe haemophilia A: Full data set from the pathfinder 3 and 5 phase III trials. *Haemophilia* 2020; 26: 450-458.

16. Fischer K, Kulkarni R, Nolan B, et al. Recombinant factor IX Fc fusion protein in children with haemophilia B (Kids B-LONG): results from a multicentre, non-randomised phase 3 study. *Lancet Haematol* 2017; 4: e75-e82.

17. Pasi KJ, Fischer K, Ragni M, et al. Long-term safety and sustained efficacy for up to 5 years of treatment with recombinant factor IX Fc fusion protein in subjects with haemophilia B: Results from the B-YOND extension study. *Haemophilia* 2020; 26: e262-e271.

18. Kenet G, Chambost H, Male C, et al. Long-acting recombinant fusion protein linking coagulation factor IX with albumin (rIX-FP) in children. Results of a phase 3 trial. *Thromb Haemost* 2016; 116: 659-668.

19. Carcao M, Zak M, Abdul Karim F, et al. Nonacog beta pegol in previously treated children with hemophilia B: results from an international open-label phase 3 trial. *J Thromb Haemost* 2016; 14: 1521-1529.

20. European Medicines Agency. Hemlibra: EPAR-Product information., <https://www.ema.europa.eu/en/medicines/human/EPAR/hemlibra#product-information-section> (2022, accessed November 2022).

21. Genetech Inc. Hemlibra® Prescribing Information, <https://www.gene.com/download/pdf/hemlibra_prescribing.pdf> (2022, accessed November 2022).

# **Supplemental Appendix. Members of the Factor Think Tank Group.**

1. Jan Astermark, Department of Translational Medicine, Lund University, and Department of Hematology, Oncology and Radiation Physics, Skåne University Hospital, Malmö, Sweden
2. Jan Blatný, Department of Pediatric Hematology, University Hospital Brno and Masaryk University, Brno, Czech Republic
3. Cristina Catarino, Immunohemotherapy Department, Hospital de Santa Maria-Centro Hospitalar Lisboa Norte, Lisbon, Portugal
4. Gerry Dolan, Centre for Haemostasis and Thrombosis, St Thomas’ Hospital, London, UK
5. Karin Fijnvandraat, Amsterdam UMC, University of Amsterdam, Emma Children's Hospital, Pediatric Hematology, Meibergdreef, Amsterdam, The Netherlands
6. Cédric Hermans, Hemostasis and Thrombosis Unit, Division of Hematology, Cliniques Universitaires Saint-Luc, Université catholique de Louvain (UCLouvain), Brussels, Belgium
7. Katharina Holstein, Department of Hematology and Oncology, University Medical Center Hamburg-Eppendorf, Hamburg, Germany
8. Victor Jiménez-Yuste, Hematology Department, Hospital Universitario La Paz, Autónoma University, Madrid, Spain
9. Robert Klamroth, Vivantes Hospital, Friedrichshain, Berlin, Germany; Institute of Experimental Hematology and Transfusion Medicine, University Hospital Bonn, Medical Faculty, University of Bonn, Bonn, Germany
10. Christoph Königs, Clinical and Molecular Hemostasis, Department of Pediatrics, University Hospital Frankfurt, Goethe University, Frankfurt, Germany
11. Michelle Lavin, Irish Centre for Vascular Biology, School of Pharmacy and Biomolecular Sciences, Royal College of Surgeons in Ireland, Dublin, Ireland; National Coagulation Centre, St James’s Hospital, Dublin, Ireland
12. Peter J. Lenting, Hémostase Inflammation Thrombose, INSERM, Unité Mixte de Recherche Scientifique 1176, Université Paris-Saclay, Le Kremlin-Bicêtre, France
13. Sébastien Lobet, Hematology Department, Saint-Luc University Clinics, Brussels, Belgium; Neuromusculoskeletal Lab (NMSK), Institute for Experimental and Clinical, Catholic University of Louvain, Woluwe-Saint-Lambert, Belgium; Physical Medicine and Rehabilitation Service, Saint-Luc University Clinics, Brussels, Belgium
14. Maria Elisa Mancuso, IRCCS Humanitas Research Hospital, Center for Thrombosis and Hemorrhagic Diseases, Rozzano, Milan, Italy
15. Jayashree Motwani, Department of Paediatric Haematology, Birmingham Children's Hospital, Birmingham, UK
16. James S. O’Donnell, National Coagulation Centre, St James’s Hospital, Dublin, Ireland; Irish Centre for Vascular Biology, School of Pharmacy and Biomolecular Sciences, Royal College of Surgeons in Ireland, Dublin, Ireland
